# Concurrent renal cell carcinoma and urothelial carcinoma: long-term follow-up study of 27 cases

**DOI:** 10.1186/s12957-018-1321-x

**Published:** 2018-01-25

**Authors:** Nienie Qi, Yue Chen, Kan Gong, Hailong Li

**Affiliations:** 1 Department of Urology, The Affiliated Hospital of Xuzhou Medical University, Xuzhou, China; 2 Department of Urology, Peking University First Hospital, Beijing, China; 3 Institute of Urology, Peking University, Beijing, China; 4 National Urological Cancer Center, Beijing, China

**Keywords:** Renal cell carcinoma, Urothelial carcinoma, Treatment, Prognosis

## Abstract

**Background:**

To investigate the clinical manifestation, diagnosis, treatment, and outcome of simultaneous occurrence of renal cell carcinoma (RCC) and urothelial carcinoma.

**Methods:**

Twenty-seven consecutive patients with synchronous renal cell carcinoma and urothelial carcinoma treated in two tertiary medical centers from March 2005 to December 2015 were retrospectively reviewed. Their clinical, pathological, and prognostic features were evaluated. Kaplan-Meier curves were used to estimate overall survival.

**Results:**

The median age was 69 years (range, 37–79 years). Seventeen patients presented with macroscopic hematuria, and 10 patients were asymptomatic. B-ultrasound, computed tomography (CT), and cystoscopy initially indicated RCC concurrent with ipsilateral upper tract urothelial carcinoma (UTUC) in 5 cases, RCC concurrent with contralateral UTUC in 1 case, RCC concurrent with bladder tumor in 17 cases, RCC concurrent with both ipsilateral UTUC and bladder tumor in 1 case, RCC in 2 cases and ureter carcinoma in 1 case. Different treatments were performed. The median follow-up time after surgery was 23 months. For patients with synchronous RCC and bladder tumor, there was no significant survival difference between patients treated with partial nephrectomy and radical nephrectomy. During follow up, four patients died of RCC, three patients died of non-oncological disease, one patient died of ureter carcinoma. The 3-year overall survival rate was 80.8%.

**Conclusions:**

Concurrence of RCC and urothelial carcinoma is clinically rare. Treatments should be individualized. The prognosis for a patient with synchronous RCC and urothelial carcinoma is possibly associated with the more aggressive one.

## Background

Renal cell carcinoma (RCC) and urothelial carcinoma are both common urological malignancies. Their simultaneous occurrence in a patient is, however, extraordinarily rare. In the English-language literature, the first case was reported by Graves and Templeton in 1921 [1]. Most studies of synchronous RCC and urothelial carcinoma have been described in case report or small series. Due to the rarity of the disease and the limited reports in the literature, the treatment and outcomes of concurrent RCC and urothelial carcinoma are still uncertain, especially for patients with concurrent RCC and bladder tumor.

In this study, we carried out a long-term follow-up of 27 cases in 2 tertiary medical centers (Peking University First Hospital and The Affiliated Hospital of Xuzhou Medical University). To the best of our knowledge, this is the largest series of concurrent renal cell carcinoma and urothelial carcinoma ever reported in English literature.

## Methods

Twenty-seven consecutive patients with synchronous RCC and urothelial carcinoma were retrospectively reviewed in this study. Informed consent was obtained from all participants. All the patients were diagnosed and treated between March 2005 and December 2015. Demographic data, including age, gender, smoking history, symptom, tumor location and size, treatment, postoperative pathological finding, adjuvant therapy, recurrence, and survival status were collected. Tumor stage was assessed according to the 2009 AJCC staging system. Tumor grade was assessed according to the WHO classification of 2004. For patients with synchronous RCC and bladder tumor, the effect of surgical type on survival was assessed by the Kaplan-Meier method and differences among survival curves were tested by the log-rank test. Among these patients, only two cases were treated with radical nephroureterectomy (RNU), so we combined these two cases with cases treated by radical nephrectomy (RN) as one group for analysis. All procedures performed in studies involving human participants were in accordance with the ethical standards of the institutional and national research committee and with the 1964 Helsinki Declaration and its later amendments or comparable ethical standards. The study was approved by the institutional review board from Peking University First Hospital and The Affiliated Hospital of Xuzhou Medical University.

## Results

### Clinical features

**Fig. 1:**
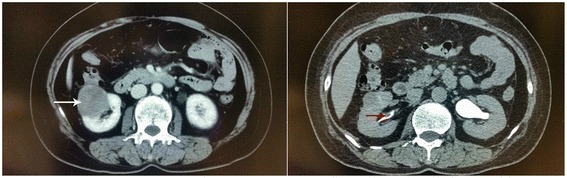
CT of a patient with concurrent renal cell carcinoma and ipsilateral pelvic carcinoma. The *white arrow* shows a renal mass in the right kidney; the *red arrow* shows a filling defect in the right renal pelvis

**Fig. 2:**
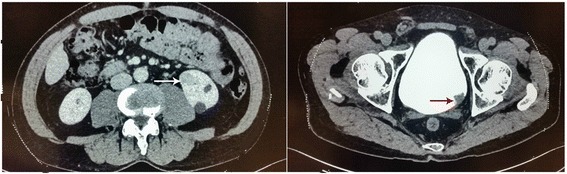
CT of a patient with concurrent renal cell carcinoma and bladder tumor. The *white arrow* shows a renal mass in the left kidney; the *red arrow* shows a filling defect in the left posterior wall of bladder

A total of 27 patients were involved in this study and patients’ characteristics are demonstrated in Table 1. The median age was 69 years (range, 37–79 years). Most of them were men. Thirteen patients (48.1%) had a smoking history. The main symptom was macroscopic hematuria, accompanied by irritative symptoms or backache. Ten patients were asymptomatic and detected by regular physical examinations. Clinical diagnosis of concurrent RCC and urothelial carcinoma was made in 24 patients (Figs. 1 and 2). Two patients were initially diagnosed as isolated RCC, and one patient was initially diagnosed as isolated ureter carcinoma.

**Table 1:** Patient and tumor demographics

	*n* = 27
Age (years), median (range)	69 (37–79)
Sex, *n* (%)	
Male	22 (81.5%)
Female	5 (18.5%)
Smoke	
Yes	13 (48.1%)
No	14 (51.9%)
Symptom	
Hematuria	17 (63.0%)
No	10 (37.0%)
UC location	
UTUC	9 (33.3%)
BT	16 (59.3%)
UTUC + BT	2 (7.4%)
RCC size (cm), median (range)	3.5 (0.6–14.5)
RCC stage	
T1	21 (77.8%)
T2	4 (14.8%)
T3	2 (7.4%)
RCC grade	
G1	8 (29.6%)
G2	16 (59.3%)
G3	3 (11.1%)
UC stage	
Ta/T1	18 (66.7%)
T2	4 (14.8%)
T3	3 (11.1%)
T2(UTUC) + T1(BT)	1 (3.7%)
T1(UTUC) + T1(BT)	1 (3.7%)
Number of tumor	
2	21 (77.8%)
3	4 (14.8%)
4	2 (7.4%)
Hydronephrosis	
Yes	7 (25.9%)
No	20 (74.1%)
Distant metastases, *n* (%)	2 (7.4%)

*UC* urothelial carcinoma, *RCC* renal cell carcinoma, *UTUC* upper tract urothelial carcinoma, *BT* bladder tumor

### Treatment and pathological findings

Table 2 reveals operative parameters, pathological findings, and follow-up results. Twenty-six patients underwent one-stage surgery, one patient underwent partial nephrectomy (PN) at first surgery due to the poor function of the contralateral kidney, and radical cystectomy (RC) was operated 1 month later. Different treatments were performed according to the tumor. Five patients diagnosed with RCC and ipsilateral upper tract urothelial carcinoma (UTUC) underwent radical nephroureterectomy; one patient diagnosed with RCC and ipsilateral UTUC and bladder tumor underwent RNU and transurethral resection of bladder tumor (TURBT); one patient diagnosed with RCC and contralateral ureter carcinoma underwent PN and contralateral ureteroureterostomy. Two patients diagnosed with metastatic renal cell carcinoma were treated with cytoreductive RN. Postoperative pathology showed these two cases were synchronous renal cell carcinoma and urothelial carcinoma in the renal pelvis. The two kinds of tumors were not intermingled, but rather separated by normal renal parenchyma, neither of the patient received further ureterectomy; one patient diagnosed with ureter carcinoma was treated with RNU, and pathological examination revealed the coexistence of ureter carcinoma and renal cell carcinoma. For patients diagnosed with RCC and bladder tumor two patients were treated with RNU and TURBT and ureter carcinoma was postoperatively found in one patient. Ten patients were treated with PN and TURBT of RC. Five patients were treated with RN and TURBT or RC. Most of the histological types of RCC were clear cell renal cell carcinomas.

**Table 2:** Operative parameters and follow-up results

	*n* = 27
Operation type	
RNU	6
RN	2
RNU + TURBT	3
PN + TURBT	9
RN + TURBT	4
PN + ureteroureterostomy	1
PN + RC	1
RN + RC	1
RCC histologic subtype, *n* (%)	
ccRCC	23 (85.2%)
pRCC	2 (7.4%)
chRCC	1 (3.7%)
Unclassified RCC	1 (3.7%)
Adjuvant therapy	
Instillation therapy	8
Radiotherapy	1
Follow-up (month), median (range)	23 (0.5–70)
Recurrence	
RCC	5
Urothelial carcinoma	7
Outcome, *n* (%)	
Alive	19 (70.4%)
Dead	8 (29.6%)

*RNU* radical nephroureterectomy, *TURBT* transurethral resection of bladder tumor, *PN* partial nephrectomy, *RN* radical nephrectomy, *RC* radical cystectomy, *ccRCC* clear cell renal cell carcinoma, *pRCC* papillary renal cell carcinoma, *chRCC* chromophobe renal cell carcinoma

### Tumor recurrence and follow-up

All the patients were regularly followed up with a median time of 23 months. During follow up, fossa recurrence of RCC appeared in one patient, bone metastasis of RCC appeared in two patients, liver metastasis of RCC appeared in one patient, and lung metastasis of RCC appeared in one patient. Bladder tumor recurrence appeared in six patients, and bone metastasis of UTUC appeared in one patient. All of the recurrence and metastasis were proved by the pathological results of surgery or biopsy. For the patient who underwent PN and contralateral ureteroureterostomy, bladder tumor occurred 1 year after initial surgery and TURBT was subsequently performed.

Seventeen patients (63.0%) survived without tumors, two patients (7.4%) survived with tumors, and eight patients (29.6%) died during the follow-up. Four patients died of RCC progress or metastases, one patient emerged with ureter carcinoma metastasis and died 3 month later, and three patients died of non-oncological disease. For patients with synchronous RCC and bladder tumor, there was no significant survival difference between patients treated with partial nephrectomy or radical nephrectomy (*P* = 0.874). The 3-year overall survival rate of all patients was 80.8%.

## Discussion

RCC represents 3% of adult cancers. Transitional cell carcinoma of the renal pelvic or ureter accounts for 5–7% of all urinary tract tumors and bladder cancer is the 11th most commonly diagnosed cancer in the world [2]. However, diagnosis of synchronous primary genitourinary tumors is uncommon. The increasing prevalence of genitourinary tumors has led to a reasonably increased diagnosis of synchronous tumors. About 50 cases of synchronous renal cell carcinoma and renal pelvic carcinoma have been reported in the literature [3–10].

The symptoms of synchronous RCC and urothelial carcinoma are similar to the solitary RCC or urothelial carcinoma of the urinary tract. In our study, hematuria was presented in 17 cases (63.0%). Of patients, 48.1% were smokers, higher than reported in literature [7]. Concurrent renal cell carcinoma and ureter carcinoma were reported in a chronic hemodialysis patient, which may imply the increased susceptibility of urological malignancy in dialysis patients [11]. However, none of them were verified as specific risk factors for the simultaneous presence of tumors.

The golden standard for management of UTUC is radical nephroureterectomy, and the best choice of treatment for patients with synchronous ipsilateral RCC and UTUC seems to be radical nephroureterectomy. However, there is no question that partial nephrectomy has become a standard to manage small renal masses. Conservative management of UTUC has been discussed in low-risk cases and allows sparing the morbidity associated with radical surgery without compromising oncological outcomes and kidney function [12]. The 2015 European Association of Urology (EAU) guidelines suggest that conservative management may be considered when the tumor is unifocal, small (< 1 cm), and low grade with no evidence of an infiltrative lesion on CT urography [13]. It therefore would appear justified to combine partial nephrectomy for RCC and kidney-sparing management for UTUC in an appropriately selected patient. Benjamin et al. have reported a patient who received renal sparing management of his double malignancy, including open partial nephrectomy of his T1a RCC and endoscopic laser ablation of his low-grade Ta ipsilateral renal pelvis urothelial carcinoma. The patient is healthy with normal serum creatinine and followed with regular surveillance ureteroscopy after 4 years [14]. This option should therefore be discussed provided that both lesions would be suitable for renal-preserving surgery. For the patient with a small renal mass (< 7 cm) and a unifocal, small (< 1 cm), and low-grade ipsilateral UTUC, partial nephrectomy of RCC and kidney-sparing management of UTUC may be combined together.

Different treatments were used to manage synchronous RCC and bladder cancer in our study. The treatment of bladder tumor was decided by tumor stage. Radical cystectomy was performed for muscle-invasive bladder tumor. For RCC, most cases were treated by partial nephrectomy. Radical nephrectomy did not improve overall survival. It is suitable to take renal-sparing surgery into consideration if RCC meets the indications for partial nephrectomy. Although none of cases with PN or with RN emerged with ureter carcinoma recurrence during follow-up, synchronous distal ureter carcinoma was detected in the pathological specimen of one patient treated with RNU. Urothelial carcinoma presents a feature of multifocality. Preoperative imaging examination should be meticulous to obtain as much information as possible and to ensure the identification of suspicious masses for patients with concurrent RCC and UC.

One patient was diagnosed as RCC and concomitant contralateral ureter carcinoma in our study. In order to avoid the risk of renal failure after PN and contralateral radical surgery, we combined bilateral renal-preserving surgery and segmental ureteral resection with uretero-ureteral anastomosis was performed for contralateral ureter carcinoma. Bladder tumor occurred after 1 year and TURBT was performed. He was still followed with regular surveillance. So far, only around 10 cases of RCC with synchronous contralateral UTUC have been described in the literature [15]. Opinions vary on proper treatment of synchronous bilateral renal tumors of different histogenesis, and no general guideline has been set. For the renal cell carcinoma, radical nephrectomy, partial nephrectomy, tumor enucleation, and angioinfarction were reported. For the renal pelvis tumor, nephroureterectomy, partial nephrectomy, radical nephrectomy. and tumor excision were reported. We believe that management plans must be individualized. All the factors including biological features of each tumor, bilateral renal function, and life quality of the patient should be considered together.

A review of 47 cases of renal tumors reported that the overall prognosis of synchronous renal tumors were not worse than isolated ones [7]. Recently, Dutta et al. have shown that the prognosis for a patient with dual malignancies is likely most influenced by the more aggressive one of the two tumors [5]. In our study, four patients died of RCC. These patients had higher stage and grade of RCC than their urothelial carcinoma, and one of them had multiple bone metastases from RCC before surgery. One patient died of ureter carcinoma 3 months after surgery with postoperative pathology of high-grade urothelial carcinoma with squamous and sarcomatous differentiation. Our result is in accordance with the hypothesis that the prognosis is possibly associated with the more aggressive one.

## Conclusions

The incidence of synchronous tumors may increase simultaneously with the increased incidence of genitourinary tumors. It is necessary to pay attention to the possible occurrence of synchronous urothelial cancer in clinical practice. Treatments should be individualized. The biological features of each tumor and bilateral renal function of the patient must be considered comprehensively. The prognosis is possibly associated with the more aggressive one. Further follow-up will combine aspects of recommended follow-up for both RCC and urothelial carcinoma, as per published guidelines.

## Abbreviations

CIS: Carcinoma in situ
CT: Computed tomography
PN: Partial nephrectomy
RC: Radical cystectomy
RCC: Renal cell carcinoma
RN: Radical nephrectomy
RNU: Radical nephroureterectomy
TURBT: Transurethral resection of bladder tumor
UTUC: Upper tract urothelial carcinoma
